# Analysis of Maternal and Fetal Oxidative Stress During Delivery with Epidural Analgesia

**DOI:** 10.1007/s43032-024-01580-1

**Published:** 2024-05-10

**Authors:** Tomoo Yuba, Yoshihisa Koyama, Yuki Kinishi, Reiko Uokawa, Chiyo Ootaki, Shoichi Shimada, Yuji Fujino

**Affiliations:** 1 Department of Anesthesiology and Intensive Care Medicine, Osaka University Graduate School of Medicine, 2-2 Yamadaoka, Suita, Osaka 565-0871 Japan; 2 Department of Neuroscience and Cell Biology, Osaka University Graduate School of Medicine, 2-2 Yamadaoka, Suita, Osaka 565-0871 Japan; 3 Addiction Research Unit, Osaka Psychiatric Research Center, Osaka Psychiatric Medical Center, Osaka, 541-8567 Japan; 4 Global Center for Medical Engineering and Informatics, Osaka University, Suita, 565-0871 Japan; 5 Integrated Frontier Research for Medical Science Division, Institute for Open and Transdisciplinary Research Initiatives (OTRI), Osaka University, Suita, 565-0871 Japan; 6 Department of Anesthesiology, Chibune General Hospital, Osaka, 555-0034 Japan

**Keywords:** Labor epidural analgesia, Oxidative stress, Fetus, Mother

## Abstract

Childbirth is a stressful event for mothers, and labor epidural analgesia (LEA) may reduce mental stress. Mental stressors include labor pain, fear, and anxiety, which induce oxidative stress. In this study, we focused on oxidative stress during delivery and conducted a cross-sectional analysis of maternal and fetal oxidative stress. The participants included 15 women who received LEA (LEA group) and 15 who did not (No LEA group). Participants with a gestational age of < 37 weeks, BMI of ≥ 35 kg/m^2^, cerebrovascular or cardiovascular complications, multiple pregnancies, gestational hypertension, gestational diabetes, chronic hypertension, thyroid disease, birth weight of < 2,500 g, emergency cesarean section, or cases in which epidural anesthesia was re-administered during delivery were excluded from the study. Maternal blood was collected on admission, and immediately after delivery, and umbilical artery blood was collected from the fetus. The oxidative stress status was assessed by measuring diacron-reactive oxygen metabolite (an index of the degree of lipid peroxide oxidation), biological antioxidant potential (an index of antioxidant capacity) and calculating the ratio of BAP/d-ROMs (an index of the oxidative stress). The results showed that maternal oxidative stress immediately after delivery was lower in the LEA group than in the No LEA group. Moreover, the fetuses experienced less oxidative stress in the LEA group than in the No LEA group. Taken together, these results suggest that LEA may reduce maternal and fetal oxidative stress associated with childbirth.

## Introduction

Labor epidural analgesia (LEA) is commonly used in painless childbirths. Epidural analgesia has been shown not to increase the cesarean section rate compared to systemic administration of analgesics and is an excellent delivery method with high satisfaction among pregnant women. LEA is also suitable for mothers with cerebrovascular or cardiovascular complications. However, the detailed effects of LEA on the mother and fetus, other than its analgesic effects, are unknown.

Reactive oxygen species (ROS) which are byproducts of respiratory metabolism, are essential for various physiological functions, such as immune responses and intracellular signaling, and are normally neutralized by in vivo antioxidants, such as superoxide dismutase 1 (SOD1) and glutathione (GSH) [1]. Oxidative stress is a state in which excess ROS generated by various causes (e.g., surfeit, long-standing disease, and viral infection) cannot be neutralized by the antioxidant mechanisms in the body, resulting in biological dysfunction due to the oxidation of biological components.

As oxidative stress is deeply involved in the pathogenesis and progression of various diseases, it is important to accurately determine the degree of oxidative stress in the body. There are two main methods for examining oxidative stress in vivo: measuring oxidants and measuring antioxidant capacity [2]. Measuring oxidants includes oxidized DNA in urine and the thiobarbituric acid reactive substances assay, which measures lipid peroxide in the blood to measure 8OHdG. Measuring antioxidant capacity includes methods for measuring the reduction capacities of SOD1 and GSH. Although this is a useful method for understanding oxidative stress because it can be performed using blood or urine, a large number of specimens are needed for analysis. Electron spin resonance spectroscopy detects free radicals, including hydroxyl radicals and superoxide radicals, in living organisms and is effective in directly measuring ROS; however, it requires special equipment and techniques, making it difficult to use in general.

Currently, the Diacron-Reactive Oxygen Metabolites (d-ROMs) and biological antioxidant potential (BAP) test is often used to analyze oxidative stress. d-ROMs reflects the amount of lipid peroxide in the blood, and its value is calculated using the oxidizing power of hydrogen peroxide as an index. BAP reflects the amount of antioxidants in the blood, and its value is calculated using the amount of iron oxide that can be reduced as an index. As aforementioned “Oxidative stress” is an unbalanced state in which the amount of oxidants exceeds the antioxidant capacity within a living body [3, 4]. Therefore, the degree of oxidative stress cannot be determined by d-ROMS or BAP values alone. In this d-ROMs and BAP test, the ratio of BAP divided by d-ROMS (BAP/d-ROMs) is used as an indicator of oxidative stress. The d-ROM and BAP test is a relatively new assay used for various diseases and has demonstrated an association between diseases and oxidative stress. d-ROMs and BAP measurements have been associated with metabolic syndrome [5] and smoking [6]. An association has also been noted in critically ill patients in the intensive care unit [7], lung cancer [8], and renal transplant patients [6].

Previous studies have shown that pregnancy and delivery have significant effects on maternal oxidative stress. In particular, delivery is a psychologically stressful event due to the pain and anxiety associated with labor [9]. Oxidative stress is also associated with psychological stressors [10]. Therefore, it is important to reduce oxidative stress associated with childbirth. However, no studies have examined the changes in oxidative stress from the onset of labor to delivery or the relationship between maternal and fetal oxidative stress. Moreover, there are currently no studies comparing the d-ROMs and BAP measured in unexposed LEA (No LEA) and LEA. In this clinical study, we analyzed the effects of LEA on the mother and fetus from the perspective of oxidative stress using d-ROMS and BAP tests.

## Methods

### Study Design

The period was from August 2022 to October 2023, and the participants were healthy pregnant women who underwent LEA (LEA group, *n* = 15) or did not undergo LEA (No LEA group, *n* = 15) at Osaka University Hospital and Chibune Hospital. This study was approved by the Ethical Review Committee of Osaka University Hospital (approval no. 22053 [T1]-3). This study was conducted in strict accordance with the principles of the Declaration of Helsinki (World Medical Association, 1964) and its subsequent revisions, which set internationally accepted ethical standards for the conduct of research involving human subjects. Participants with a gestational age of < 37 weeks, BMI ≥ 35 kg/m^2^, cerebrovascular or cardiovascular complications, multiple pregnancies, gestational hypertension, gestational diabetes, chronic hypertension, thyroid disease, birth weight of < 2,500 g, emergency cesarean section, or cases in which epidural anesthesia was re-administered during delivery were excluded from the study. In obesity and diabetes, fat and systemic ROS derived from mast adipocytes, and unfavorable lifestyle habits are associated with increased NADPH-oxidase and suppression of antioxidant enzymes, affecting oxidative stress [5]. Because this research focuses on analysis of oxidative stress related to pregnancy and childbirth, items that affect oxidative stress, such as obesity and diabetes, are excluded. After hospitalization for labor pain, a pre-partum blood sample was drawn at the time of transfer to the delivery room, and a post-partum blood sample was drawn 1 h after delivery. In this study, the decision to move pregnant woman to the delivery room was made when regular contractions occurred approximately every 3 min and further labor progression was anticipated. At that time, the cervical dilation was approximately 2 to 3 cm. Similarly, the timing of the initiation of LEA largely coincided with that of above-mentioned moving, although LEA might be administered at pregnant request if they were still able to tolerate the pain and wished to wait. Fetal blood specimens were obtained from surplus samples from umbilical artery blood tests routinely performed at birth. Blood samples were centrifuged at 4 °C for 10 min at 3,000 rpm immediately after collection, and the supernatant was stored at -30 °C until immediately before measurement. The basic information (Table 1), such as height, weight, age, and medical history, information related to pregnancy and delivery (pregnancy history, delivery history, gestational age, birth weight, duration of the first and second stages of labor, Apgar score, umbilical artery blood pH, and umbilical artery blood sBE), and cervical dilation if LEA was performed, were extracted from electronic medical records. The primary endpoint was whether maternal and fetal oxidative stress levels differed with or without LEA, and the secondary endpoint was the identification of other factors associated with oxidative stress levels.

**Table 1:**
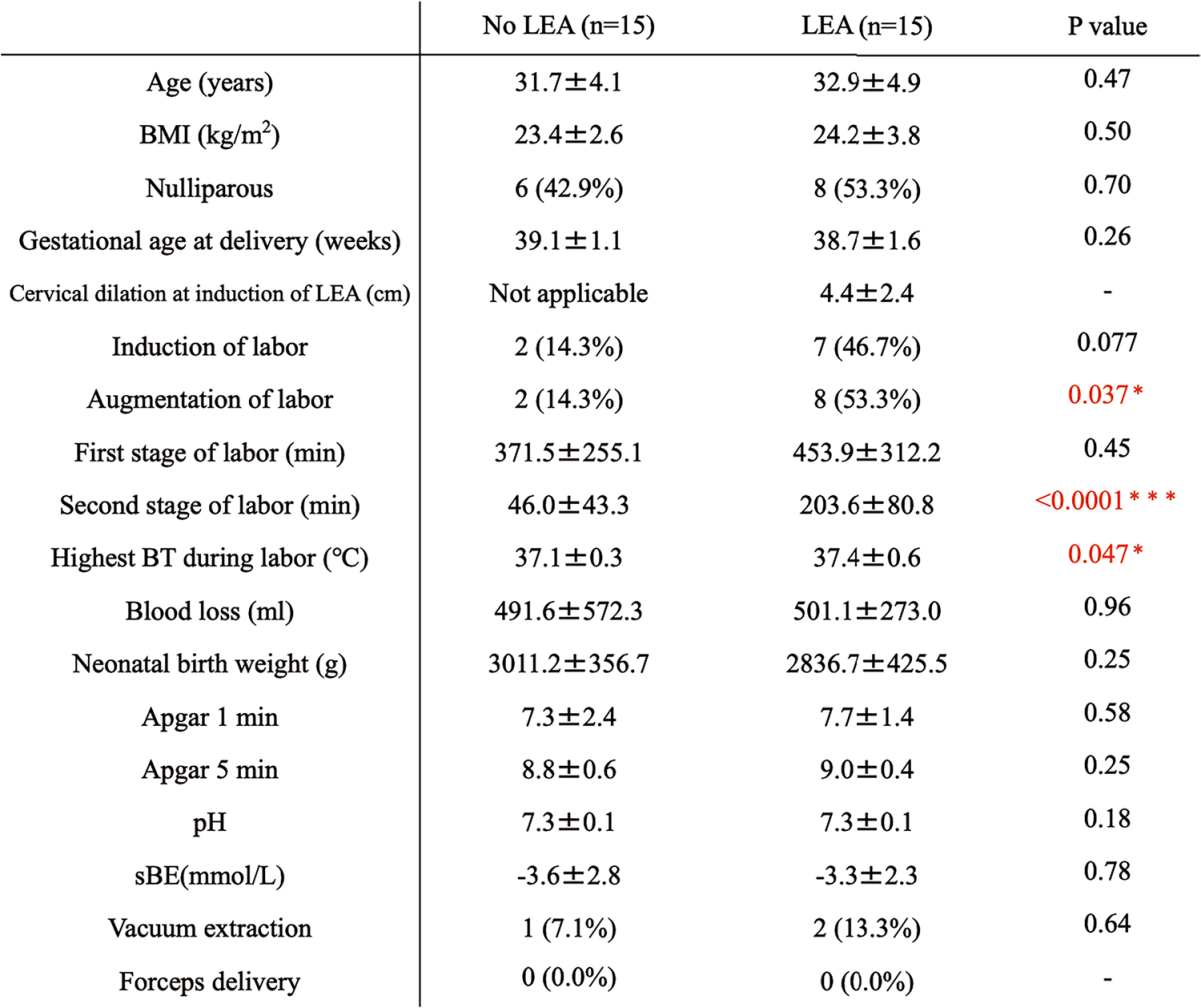
Pregnant background of No LEA (*n* = 15) and LEA (*n* = 15) groups. Red: Significant difference. Data are expressed as mean ± SEM or N (%). *p < 0.05, ***p < 0.0001 vs. No LEA group, determined by Student's paired *t*-test

### Oxidative Stress Measurement

To investigate the serum levels of ROS metabolites and antioxidant capacity, the levels of d-ROMs and BAP were measured using a REDOXLIBLA (Wismerll Co. Ltd., Tokyo, Japan). The results of d-ROM test were expressed in Carratelli units (U CARR); 1 U CARR corresponds to 0.8 mg/L of hydrogen peroxide. BAP indicated the reducing power of blood using the amount of trivalent iron ions (μM) reduced to divalent iron ions as an indicator. Comparative analysis was performed using the d-ROM value, BAP, and the ratio of BAP/d-ROMs.

### Statistical Analysis

Data are expressed as mean ± standard deviation or numerical values (%). Continuous variables were compared using Student's t-test or Mann–Whitney's U-test, and categorical variables were compared using the χ^2^ or Fisher's exact tests. Spearman's rank correlation coefficient was used to determine the correlation between two continuous variables. For all analyses, ^*^p < 0.05 and ^♯^*p* < 0.08, was considered statistically significant (^*^p) or as a tendency (^♯^*p*).

## Results

**Fig. 1:**
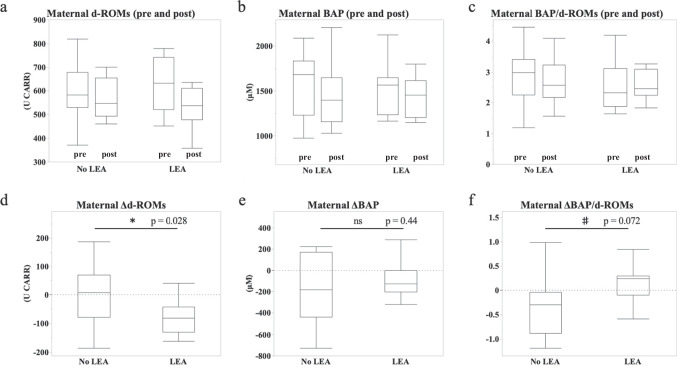
**a**-**c**: Bar graphs of maternal d-ROMs (**a**), BAP (**b**), and BAP/d-ROMs (**c**) before (left) and after delivery (right). **d**-**f**: Bar graphs of maternal d-ROMs (**d**), BAP (**e**), and BAP/d-ROMs (**f**) before delivery versus the amount of change after delivery. No LEA group (*n* = 15); LEA group (*n* = 15). The boundary of the box closest to zero indicates the 25th percentile, the line within the box marks the median, and the boundary of the box farthest from zero indicates the 75th percentile. Whiskers (error bars) above and below the box indicate the 90th and 10th percentiles. *p < 0.05, ^♯^*p* < 0.08 vs. No LEA group, determined by Student’s paired *t*-test. ns: not significant

Maternal pre-partum d-ROMs and BAP were 590.0 ± 121.8 U CARR, 1610.0 ± 347.8 μM in the No LEA group, and 622.9 ± 108.4 U CARR, 1523.6 ± 285.1 μM in the LEA group respectively, with no difference between two groups (Fig. 1a left, 1b left). After delivery, maternal d-ROMs of the LEA group tended to be lower than that of the No LEA group (Fig. 1a Right; No LEA 575.3 ± 90.0 U CARR, LEA 535.7 ± 80.5 U CARR), but there was no significant difference in BAP between two groups (Fig. 1b left: No LEA 1610.1 ± 347.8 μM, LEA 1523.6 ± 285.1 μM; Fig. 1b right: No LEA 1461.8 ± 329.5 μM, LEA 1444.6 ± 215.1 μM). Maternal BAP/d-ROMs before and after delivery were not different between the two groups (Fig. 1c left: No LEA 2.87 ± 0.91, LEA 2.55 ± 0.78; Fig. 1c right: No LEA 2.64 ± 0.78, LEA 2.70 ± 0.79). To consider the individual differences in each item, we analyzed the amount of change in each item before and after delivery. The change in maternal d-ROMs before and after delivery was -15.6 ± 82.8 U CARR in the No LEA group and -85.0 ± 61.3 U CARR in the LEA group, with a predominant decrease in the LEA group (Fig. 1d, *p* = 0.028). The changes in BAP and BAP/d-ROMs tended to be larger in the LEA group than in the No LEA group (Fig. 1e: BAP. No LEA -148.3 ± 307.5 μM, LEA -79.0 ± 170.7 μM; Fig. 1f: BAP/d-ROMs. No LEA -0.23 ± 0.65, LEA 0.14 ± 0.53). These results indicate that maternal oxidative stress was alleviated by LEA.

**Fig. 2:**
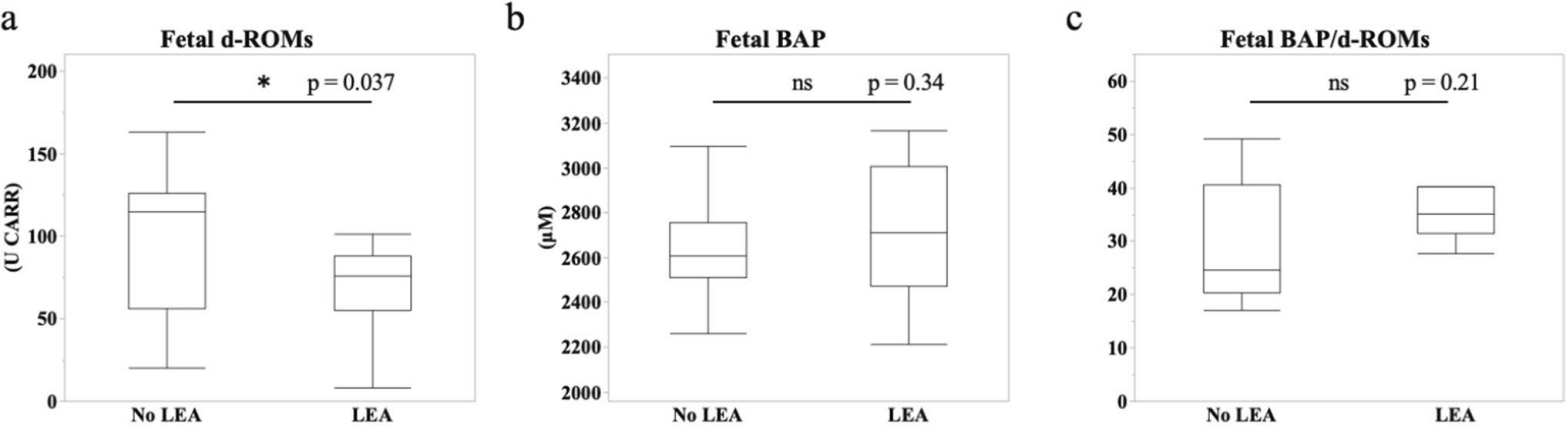
Bar graphs of fetal d-ROMs (**a**), BAP (**b**), and BAP/d-ROMs (**c**) immediately after birth by umbilical artery blood. No LEA group (*n* = 15); LEA group (*n* = 15). The boundary of the box closest to zero indicates the 25th percentile, the line within the box marks the median, and the boundary of the box farthest from zero indicates the 75th percentile. Whiskers (error bars) above and below the box indicate the 90th and 10th percentiles. *p < 0.05 vs. No LEA group, determined by Student's paired *t*-test. ns: not significant

**Fig. 3:**
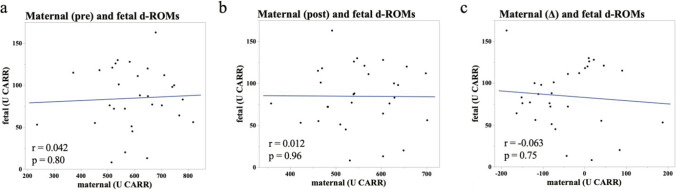
**a**-**c**: Relationship between fetal d-ROMs and maternal d-ROMs of pre-partum (**a**), post-partum (**b**), and periparturient change (**c**). The blue line is an approximate formula. Spearman's rank correlation coefficient was used to correlate fetal and maternal d-ROMs

Next, we investigated fetal oxidative stress using umbilical artery blood. Fetal d-ROMs were 103.5 ± 42.5 U CARR in the No LEA group and 71.0 ± 28.9 U CARR in the LEA group and were significantly lower in the LEA group as compared with No LEA group (Fig. 2a, *p* = 0.037). Although there were no significant differences in fetal BAP and BAP/d-ROMs between the two groups, fetal BAP/d-ROMs tended to be higher in the LEA group than in the No LEA group (Fig. 2b: BAP. No LEA 2595.9 ± 243.5 μM, LEA 2711.5 ± 301.0 μM; Fig. 2c: BAP/d-ROMs. No LEA 36.57 ± 31.75, LEA 69.81 ± 98.92). Maternal pre- and post-partum d-ROMs and changes in d-ROMs were not related to fetal d-ROMs (Fig. 3a-c; 3a: vs. pre-partum, *r* = 0.045, *p* = 0.80; 3b: vs. post-partum, *r* = -0.012, *p* = 0.96; 3c: vs. change, *r* = -0.063, *p* = 0.75). These findings suggested that LEA alleviated fetal oxidative stress. The results for both the mother and fetus are summarized in Table 2.

**Table 2:**
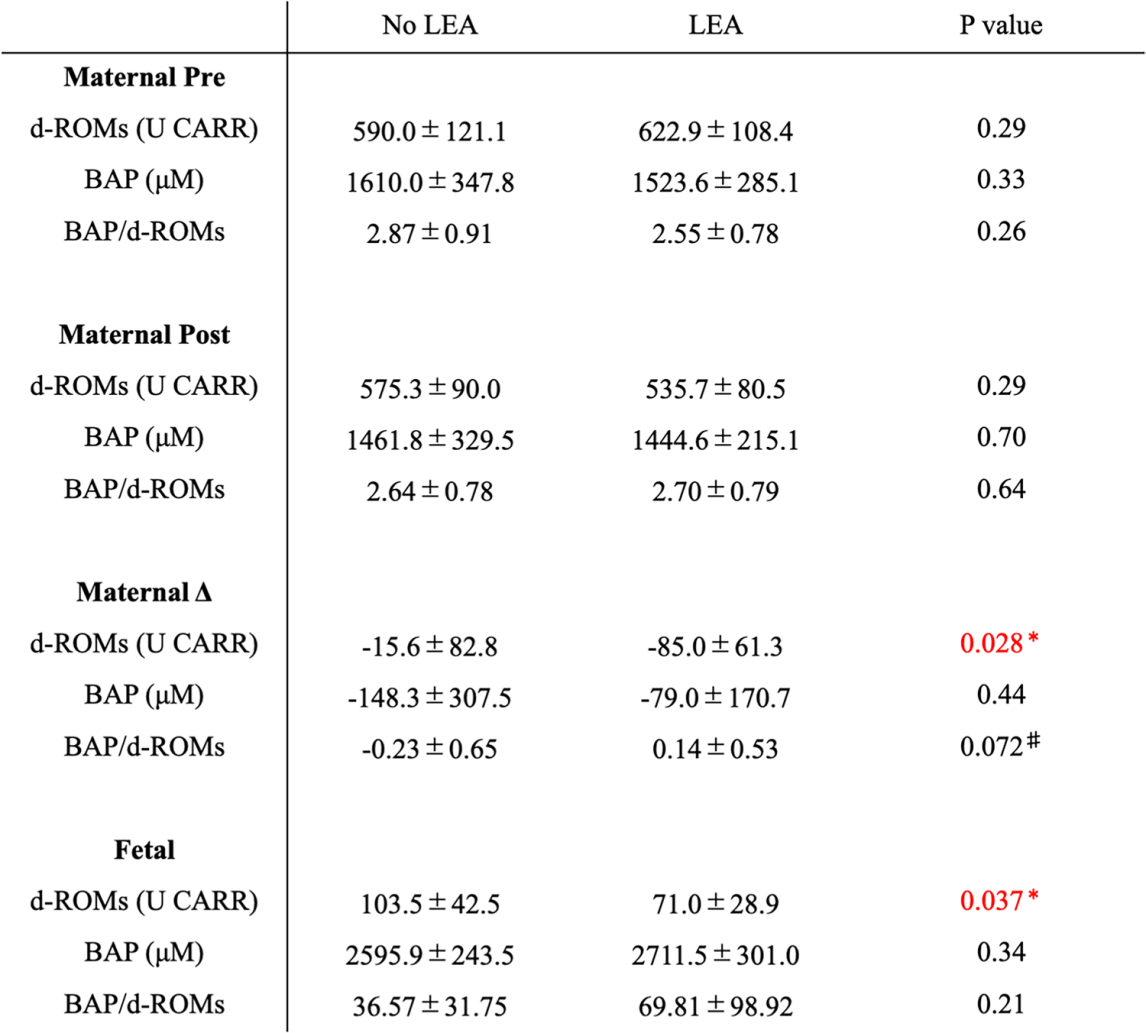
Results of No LEA (*n* = 15) and LEA (*n* = 15) groups. Red: Significant difference. Data are expressed as mean ± SEM. *p < 0.05, ^♯^*p* < 0.08 vs. No LEA group, determined by Student’s paired *t*-test

**Fig. 4:**
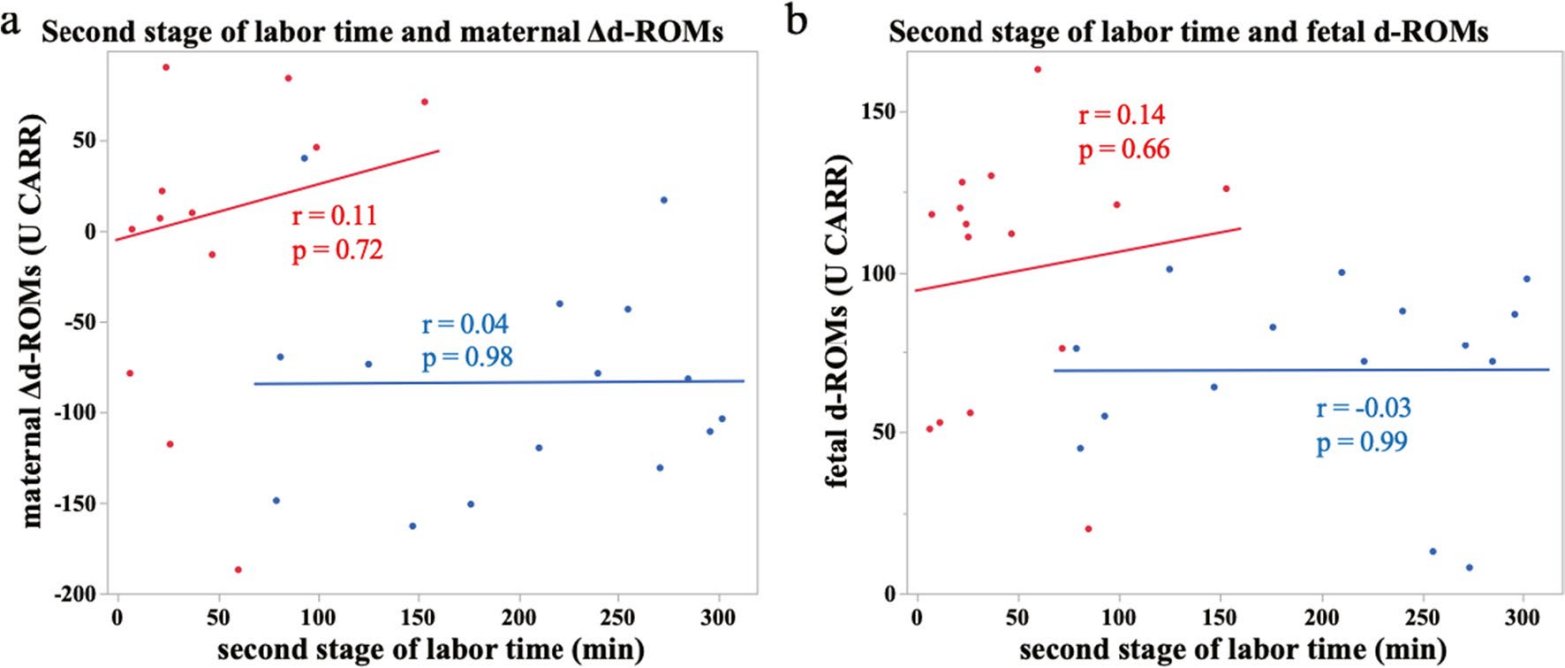
**a**, **b**: Relationship between time of the second stage of delivery and the amount of change in maternal d-ROMs (**a**) and fetal d-ROMs (**b**). Approximate lines: red (No LEA group, *n* = 15), blue (LEA group, *n* = 15)

Moreover, we investigated whether there was a correlation between the time of the second stage of labor and the amount of change in maternal or fetal d-ROMs before and after delivery. There was no association in either case (Fig. 4a, 4b; 4a: vs. LEA d-ROM change *r* = 0.04, *p* = 0.98, vs. No LEA d-ROM change *r* = 0.11, *p* = 0.72, 4b: vs. LEA fetal d-ROM *r* = -0.03, *p* = 0.99, vs. No LEA fetal d-ROM *r* = 0.14, *p* = 0.66). However, d-ROMs increased with time in the second stage of labor in the NO LEA group but not in the LEA group. Taken together, these results suggest that the effects of LEA on reducing oxidative stress and prolonging the second stage of labor may be independent events.

**Fig. 5:**
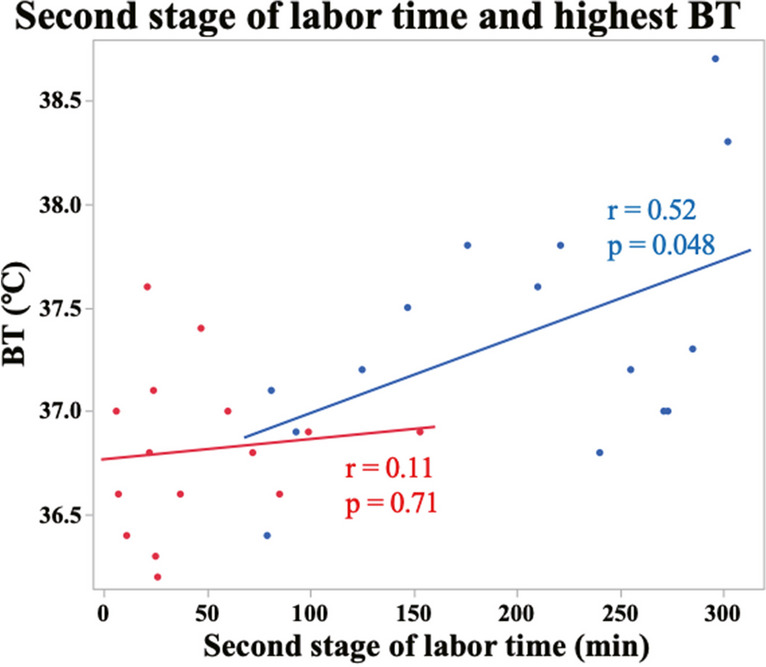
Relationship between the second stage of labor and maximum body temperature during labor. Approximate lines: Red (No LEA group, *n* = 15), blue (LEA group, *n* = 15). Spearman's rank correlation coefficient was used to correlate the temperature and delivery time in the No LEA and LEA groups

**Fig. 6:**
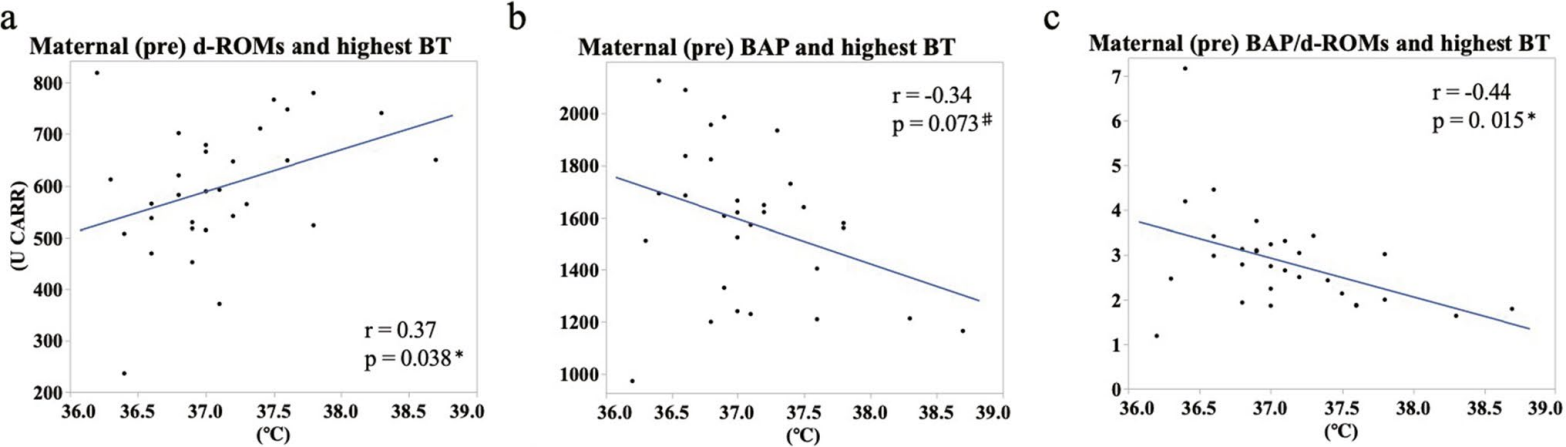
Relationship between the prepartum value of oxidative stress and maximum body temperature during labor. **a**: d-ROMs, **b**: BAP and **c**: BAP/d-ROMs. Spearman's rank correlation coefficient was used to correlate the temperature and the Prepartum value. *p < 0.05, ^♯^*p* < 0.08

Finally, the pregnancy background and obstetric outcomes of the No LEA and LEA groups are shown in Table 1. The LEA group had significantly more labor augmentation, longer second stage of labor time, and higher maximum body temperature than the no-LEA group (Table 1, augmentation of labor, *p* = 0.037; second stage of labor time, *p* < 0.0001; maximum body temperature, *p* = 0.047). The highest temperature during delivery was not associated with the duration of the second stage of labor in the No LEA group but with the duration of the second stage of labor in the LEA group (Fig. 5. No LEA: *r* = 0.11, *p* = 0.71; LEA: *r* = 0.52 *p* = 0.048). Moreover, we analyzed the correlation between pre and post levels of d-ROMs, BAP, BAP/d-ROMs, the change in these markers, and the maximum body temperature during labor among all 30 participants, regardless of whether they received LEA. As a result, significant correlations were found between antepartum d-ROMs and BAP/d-ROMs ratios (Fig. 6. *p* = 0.038 and *p* = 0.015, respectively). Furthermore, a correlation trend was observed for antepartum BAP levels (*p* = 0.073). Although not statistically significant, the No LEA group tended to have more induced deliveries (*p* = 0.077).

## Discussion

LEA reduced maternal and fetal oxidative stress compared with No LEA. This suggests that LEA has other effects besides analgesia in reducing oxidative stress during delivery and may prevent excessive oxidative stress.

Several previous reports have shown that oxidative stress is increased in pregnant women. Lucie et al. found that urinary 8-hydroxydeoxyguanosine (8-OHdG), an oxidized derivative of guanosine (oxidative stress biomarker), is increased in pregnant women [11]. Although d-ROMs and BAP values vary from individual to individual, the d-ROMs and BAP of non-pregnant women were approximately 300–350 U CARR and about 2,000–2,500 μM, respectively [12]. In the present study, the oxidative stress values of the pregnant women were also high, and their antioxidant capacity was also decreased (No LEA group: d-ROMs 590.0 ± 121.8 U CARR, BAP 1610.0 ± 347.8 μM; LEA group: d-ROMs 622.9 ± 108.4 U CARR, BAP 1523.6 ± 285.1 μM). At 16–30 weeks of gestation, maternal d-ROMs increase, BAP decreases, and oxidative stress increases with increasing gestational age [13]. The levels of d-ROMs and BAP at 36 weeks of gestation [14] were almost the same as those before maternal delivery in this study, suggesting little increase in oxidative stress after 36 weeks. Increased maternal oxidative stress during pregnancy is believed to be related to increases in cardiac output and circulating blood volume, blood gas exchange, and oxygen consumption [13]. To date, there are several papers investigated the effects of LEA on pregnancy-related oxidative stress. According to Idris et al., maternal malondialdehyde levels at delivery were lower in the LEA group than in the No LEA group [15], and LEA reduced LP, GSH, and CAT [16]. However, since these studies only measured either oxides or antioxidants, it is difficult to determine whether LEA actually reduces oxidative stress. Even if oxides increase, if antioxidants also increase, oxidative stress may not increase. Conversely, even if oxidants decrease, if antioxidants decrease, oxidative stress may not decrease. “Oxidative stress” is a state of quantitative imbalance between oxides and antioxidants, so it is very important to measure both oxides and antioxidants to accurately analyze oxidative stress. Our results revealed that LEA significantly decreased maternal d-ROMs levels (oxidant levels) but did not affect BAP levels (antioxidant levels) (Fig. 1d, 1e). Additionally, we found that LEA tended to reduce oxidative stress (Fig. 1f). It has been previously reported that maternal oxidative stress levels decrease postpartum, as evidenced by previous studies [17]. The observed reduction in postpartum oxidative metabolites might be attributed to the process of childbirth and the expulsion of the placenta. However, in this study, blood samples were not collected during labor, so the detailed change in oxidative stress from labor to delivery remain unknown. Therefore, it is difficult to determine whether the decrease in oxidative stress in the LEA group after delivery was caused by the alleviation of the increased oxidative stress associated with delivery or the enhancement of the mitigation effect of oxidization. To accurately capture the detailed changes in oxidative stress levels during delivery and the effects of LEA, it is necessary to consider blood sampling during delivery. In summary, it was suggested that the reduction of oxides might be a major contributor to the reduction of oxidative stress associated with pregnancy due to LEA.

LEA is a method of achieving maternal satisfaction mainly by reducing pain associated with childbirth; however, this study demonstrated that LEA reduced the elevated oxidative stress of the mother associated with childbirth. Prolonged exposure to intense pain might also have an effect. Aerobic respiration is one factor that increases oxidative stress, particularly in the presence of pain and anxiety [18]. LEA may reduce excessive respiration and elevate maternal oxidative stress by reducing pain and anxiety. Reportedly, gestational hypertension and gestational diabetes mellitus have also been reported to be associated with oxidative stress in their pathogenesis [19, 20]. In this way, oxidative stress during pregnancy has been linked to various childbirth-related diseases, so it would be better to prevent oxidative stress from becoming excessive during pregnancy. Unfortunately, it is still unclear whether alleviation of oxidative stress during parturition can reduce the onset of these diseases. However, as oxidative stress during delivery is also part of the oxidative stress associated with pregnancy, it is possible that LEA may reduce the incidence of childbirth-related diseases.

An imbalance between inflammation and oxidative stress is associated with placental abnormalities [19]. They are implicated in placental angiogenesis defects such as eclampsia; therefore, it is important to alleviate excessive oxidative stress during pregnancy, especially in the placenta [21]. According to previous reports, excessive oxidative stress greatly affects postnatal brain development, leading to mental disorders. Micangeli et al. reported that oxidative stress caused by ethanol consumption during pregnancy may be one possible factor in causing genetic changes associated with Down syndrome and autism spectrum disorders [22]. Moreover, maternal immune activation is a risk factor for schizophrenia and autism, and oxidative stress has been suggested as one of the many factors affecting the downregulation of a group of genes associated with autism in a study using rats [23]. Therefore, reduction of fetal oxidative stress might reduce the incidence of mental disorders related to oxidative stress [24]. In fact, intake of antioxidants such as vitamin C during pregnancy is associated with a lower risk of developing autism spectrum disorders in newborns. Additionally, in a Norwegian mother-infant cohort, maternal folic acid supplementation during early pregnancy was associated with a lower risk of severe language delay in children at 3 years of age. [25]. There are few reports comparing fetal oxidative stress between LEA and No LEA. Zita et al. reported lower SOD levels in umbilical artery blood from LEA patients compared to those from patients with No LEA [16]. Additionally, there was also report that LEA reduced oxides such as 8-OHdG and 4-hydroxy-2-nonenal in the placenta [26]. Our results also revealed the reduction of oxides by LEA (Fig. 2a). Notably, LEA alleviated the increase in both maternal and fetal oxide despite a prolonged second stage of labor. Conversely, maternal and fetal oxidative stress tended to increase in proportion to the second stage of labor (Fig. 4). LEA was found to be highly effective in reducing oxidative stress associated with childbirth. However, it is still unclear how reducing oxidative stress during delivery affects the development of childbirth-related diseases. Therefore, future research will be necessary to follow the development of children born through LEA.

There were large differences in the magnitude of d-ROMs and BAP values between mothers and fetuses, and maternal oxidative stress at birth was not associated with fetal oxidative stress in any of the evaluation items (Fig. 3). The fetus is highly vulnerable to oxidative stress. The placenta maintains the fetus in a low-oxygen environment, which protects it from ROS. Furthermore, fetuses are exposed to a relatively low-oxygen environment in the uterus, which may make them less susceptible to oxidative stress resulting from aerobic respiration. Moreover, amniotic fluid has antioxidant properties, and the fetus is protected from oxidative stress by the placenta and amniotic fluid [27, 28]. Therefore, it is natural that there is a discrepancy between maternal and fetal values. However, considering the permeability of the placenta, it is possible that maternal oxidative stress may affect fetal oxidative stress. Animal experiments using labeled fatty acids had also shown that fatty acids cross the placenta [29]. Moreover, according to a clinical research report that investigated the permeability of lipids in the placenta, unsaturated fatty acids such as arachidonic acid permeate the placenta more easily than saturated fatty acids, and unsaturated fatty acids are oxidized to lipid peroxides [30]. Therefore, we thought that lipid peroxide in the mother's blood has a high possibility of affecting the fetus through the placenta. Especially, the placenta, which has been damaged by excessive oxidative stress, has increased permeability, and there is a high possibility that maternal oxidants can reach the fetus. Unfortunately, there are no reports on discrepancies between maternal and fetal oxidative stress values. In the present study, it revealed that the fetus of a mother with high oxidative stress does not necessarily have high oxidative stress (Fig. 2). These findings suggest that the fetus and mother might have separate environments across the placenta and that oxidative stress in the fetus may not affect the increase or decrease in oxidative stress in a normal mother. To compare oxidative stress between mothers and children, it is necessary to increase the number of cases and analyze the postnatal course of the fetus.

The LEA group had significantly more labor augmentation and longer second stages than the No LEA group (Table 1, augmentation of labor, *p* = 0.037; second stage of labor, *p* < 0.0001). Although not statistically significant, the No LEA group tended to have more induced deliveries (*p* = 0.077). Various reports have shown that delivery time is longer in LEA, necessitating the use of drugs to accelerate delivery [31]. The results of the present study were similar to those of previous reports, with LEA showing a prolonged delivery time and increased frequency of oxytocin use. In contrast, the LEA group had significantly higher maximum body temperatures than the No LEA group (Table 1, maximum body temperature, *p* = 0.047). The highest temperature during delivery was associated with the duration of the second stage of labor in the LEA group but not in the No LEA group (Fig. 5, *p* = 0.032). Previous reports have shown that LEA increases maternal fever during labor, but the underlying mechanism remains unknown [31, 32]. The observation in this study of a correlation between fever during labor and prepartum oxidative stress levels may contribute to our understanding of fever associated with childbirth. It suggests that not pregnant woman receiving LEA develop fever, and this variability might be anticipated by the levels of oxidative stress before labor. In other words, it was revealed that reducing oxidative stress is important for preventing fever associated with LEA. Moreover, non-infectious fever associated with LEA is not expected to affect the fetus, but it is difficult to determine if the fever is infectious or not; therefore, it is important to try to shorten the delivery time as much as possible by using antibiotics and oxytocin if fever is observed during delivery [32]. Our results showed that the LEA group was more likely to have a fever, and they also used oxytocin to shorten the delivery time as much as possible (Table 1). Epidural anesthesia generally tends to lower a patient’s temperature [33]; however, when epidural anesthesia is administered to a pregnant woman, it tends to increase her temperature. As in many other studies [31, 32], maternal temperatures tended to increase in the LEA group in this study; the longer the second stage of labor in the LEA group, the higher the fever (Fig. 5). In line with our findings, Goetzl et al. also revealed that mothers who developed a fever exhibited significantly higher levels of oxidative stress compared to those who did not experience a fever [34]. Our study also revealed a significant decrease in oxidative stress and increase in body temperature in the LEA group compared to the NO LEA group. Although it is currently not well understood about fever and oxidative stress during childbirth, these findings suggest that there may be a relationship between the two. Further studies are required to investigate the role of LEA in body temperature regulation.

The timing of maternal blood collection before delivery was when the mother was moved to the delivery room; however, the progress of delivery was not always the same in each case; the timing of maternal blood collection after delivery was generally 30–60 min, but not at the same time. The amount of oxytocin used was also unknown, and the timing of the temperature measurements varied from mother to mother. It is extremely difficult to collect blood from mothers before and after delivery; therefore, these points are the limitations of present study.

This study showed that LEA reduced oxidative stress in the mother and fetus. A certain amount of oxidative stress is necessary for survival, and it is unclear what level of oxidative stress is appropriate for mothers and fetuses under the special circumstances of pregnancy and delivery. However, an increase in oxidative stress associated with delivery should be avoided in both the mother and fetus. In particular, considering the various postnatal effects of excessive oxidative stress on the fetus, which is vulnerable to oxidative stress, the increase in oxidative stress associated with childbirth should be minimized as much as possible. We believe that LEA is an excellent method that provides multiple benefits to the mother and fetus, in addition to analgesia, as long as the medical staff administer it safely. Our study reveals effects of LEA on the mother and fetus besides analgesia, suggests the existence of an independent antioxidant system in the fetus. We hope that future research will reveal the significance of reducing oxidative stress through LEA.

## Data Availability

All relevant data are within the paper and its Supplementary Information files.

## Declarations

This study was conducted in strict accordance with the principles of the Declaration of Helsinki (World Medical Association, 1964) and its subsequent revisions, which set internationally accepted ethical standards for the conduct of research involving human subjects.
